# The value of repeat radial-probe endobronchial ultrasound-guided transbronchial biopsy after initial non-diagnostic results in patients with peripheral pulmonary lesions

**DOI:** 10.1186/s12890-017-0478-3

**Published:** 2017-10-17

**Authors:** Chun-Ta Huang, Yi-Ju Tsai, Chao-Chi Ho, Chong-Jen Yu

**Affiliations:** 1 Department of Internal Medicine, National Taiwan University Hospital, No. 7 Chung-Shan South Rd, Taipei, 100 Taiwan; 2 Department of Traumatology, National Taiwan University Hospital, Taipei, Taiwan; 3 Graduate Institute of Clinical Medicine, College of Medicine, National Taiwan University, Taipei, Taiwan; 4 Graduate Institute of Biomedical and Pharmaceutical Science, College of Medicine, Fu Jen Catholic University, New Taipei City, Taiwan

**Keywords:** Biopsy, Bronchoscopy, Diagnosis, Endobronchial ultrasound, Peripheral pulmonary lesion, Radial probe

## Abstract

**Background:**

Radial-probe endobronchial ultrasound (rEBUS)-guided transbronchial biopsy (TBB) is invaluable in the diagnosis of peripheral pulmonary lesions (PPLs); however, in certain instances, the procedure has to be repeated because of initial non-diagnostic procedure(s). Little if any literature has been published on this issue. Therefore, the aim of this study was to investigate the utility of repeat rEBUS-guided TBB in achieving a definitive diagnosis of PPLs.

**Methods:**

All patients who underwent rEBUS-guided TBB of PPLs at National Taiwan University Hospital between 2011 and 2015 and had a repeat procedure after non-diagnostic initial procedures were identified as the study subjects. The primary outcome of interest was the diagnostic yield of repeat rEBUS-guided TBB for PPLs. Also, we sought to discover features associated with the yield of repeat procedures.

**Results:**

Forty-three (11%) out of 384 patients with initial non-diagnostic TBB were included for analysis. A diagnosis of PPLs was able to be confirmed with repeat TBB in 23(53%) patients. The pathology of the first TBB was significantly associated with the yield of repeat procedures (*P* = 0.011). Further, patients with normal lung tissue in initial pathology rarely (2/12, 17%) had a definite diagnosis on repeat TBB. Yet, patients with pathology showing atypical cells and other non-specific findings were more likely (21/31, 68%) to obtain a confirmed diagnosis. The diagnostic yield of repeat procedures was not affected by the size, location or CT appearance of the lesions, or position of the rEBUS probe. No death or other serious adverse events occurred with the repeat rEBUS-guided procedures.

**Conclusions:**

If clinically indicated, it is reasonable to repeat rEBUS-guided TBB after an initial non-diagnostic procedure as the diagnostic yield will be at least 50% and the side effect profile is favorable.

## Background

Radial-probe endobronchial ultrasound (rEBUS)-guided transbronchial biopsy (TBB) is a minimally invasive diagnostic procedure for lung cancer and other lung diseases. Since its advent in 2002, [1] it has been adopted worldwide to increase the diagnostic yield of peripheral pulmonary lesions (PPLs) and has supplanted conventional TBB [2]. In addition, the favorable safety profile and fair diagnostic yield make rEBUS-guided TBB one of the preferred diagnostic modality for PPLs [3–5]. However, a non-diagnostic result in rEBUS-guided TBB for PPLs is not uncommon and may be observed in as high as around 50% of procedures of this type [6, 7]. Further investigations, such as surgery, CT-guided biopsy or clinical follow-up, should be considered with regard to those lesions to achieve a definitive diagnosis.

In clinical practice, repeat rEBUS-guided TBB may also be a viable option after initial non-diagnostic procedures for PPLs. Nevertheless, to the best of our knowledge, little if any literature concerning this issue has been published. It is important to understand the utility of repeat rEBUS-guided TBB procedures during the diagnostic process for PPLs because this information will help clinicians to schedule individualized diagnostic plans for each of the patients. In this study, we aimed to assess the diagnostic yield of repeat rEBUS-guided TBB to better define its efficacy and to identify clinical features associated with the yield of repeat procedures. Also, we proposed some indications for repeat rEBUS-guided TBB in the diagnosis of PPLs.

## Methods

### Study setting and subjects

This study was conducted at National Taiwan University Hospital, a tertiary-care referral center in Northern Taiwan. From the bronchoscopy registry, patients having 2 or more sessions of rEBUS-guided TBB during the 5-year period of 2011 through 2015 were identified. Patients were included if they were aged 20 or more and required repeat procedures to obtain a final diagnosis of the same PPLs. No exclusion criteria were applied in this study. The decision to choose rEBUS-guided TBB as the second diagnostic procedure was made at the discretion of the referring physician(s) and no specific predefined criteria were set for this during the study period. Approval was obtained from the Research Ethics Committee of National Taiwan University Hospital prior to any data collection and analysis taking place in this study, and written informed consent was obtained before each bronchoscopic procedure.

### The rEBUS-guided TBB procedures

The rEBUS procedures were performed with an endoscopic ultrasound center (EU-M30S; Olympus) and a 20-MHz radial-type ultrasonic probe (UM-S20-20R; Olympus). Bronchoscopic procedures were conducted using local anesthesia with lidocaine and intramuscular meperidine if not contraindicated by supervised chest fellows. No consciousness sedation was applied throughout the study period. Pulse oximetry was used to monitor oxygenation during the procedure and oxygen was administered via a nasal prong whenever required to maintain oxygen saturation > 90%. Under rEBUS guidance, TBB was performed in a manner similar to that previously described. [7] In brief, after localizing the PPL on the rEBUS image, the distance between the bronchial orifice and the lesion was determined. The rEBUS probe was then withdrawn and the biopsy forceps was introduced into the working channel of the bronchoscope. The forceps was advanced into the bronchus to the predetermined distance until the cusp was expected to reach the lesion, and the forceps was then closed and retracted. The procedure was repeated until two adequate specimens, defined as lung specimens spilling over the surface of the biopsy forceps, were collected. Bronchial washing or brushing was performed at the same setting depending on the discretion of the responsible pulmonologist(s). Because of the superior safety profile of rEBUS-guided TBB, a chest radiograph was only obtained as clinically indicated.

### Retrieved data

Information extracted from patient charts included patient demographics, clinical characteristics, procedural information, adverse events, initial non-diagnostic pathology, time interval between repeat rEBUS-guided TBB procedures, and final diagnoses of the PPLs. The rEBUS probe position was classified as either within or adjacent to the PPLs as previously described [8]. The rEBUS-guided procedures were considered diagnostic only if a specific malignant or benign diagnosis of PPLs was made. Patients not diagnosed after repeat rEBUS-guided TBB were subjected to other diagnostic modalities, or clinical or radiographical follow-up for a definitive diagnosis. The PPLs were deemed benign if their sizes were either stationary or diminished for at least one-year follow-up.

### Statistical analyses

The primary outcome was the diagnostic yield of repeat rEBUS-guided TBB for PPLs. The continuous variables were expressed as means ± standard deviations and were compared using Student’s t testing. The categorical variables were reported as a number (percentage) and were compared using the χ^2^ or Fisher’s exact test as appropriate. The statistical analyses were performed using SPSS statistical software (SPSS version 12.0, Chicago, IL). All of the tests were two-tailed and a *P* value of <0.05 indicated statistical significance.

## Results

### Study population

**Fig. 1:**
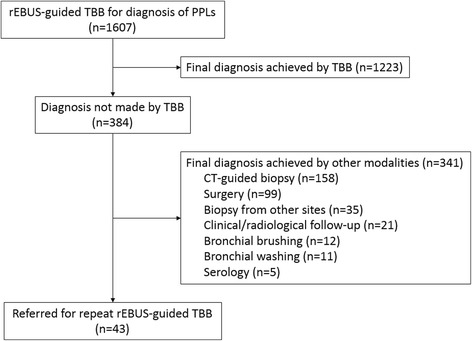
Study flow diagram. CT, computed tomography; rEBUS, radial-probe endobronchial ultrasound; PPL, peripheral pulmonary lesion; TBB, transbronchial biopsy

Between January 2011 and December 2015, a total of 1607 patients underwent rEBUS-guided TBB for the diagnosis of PPLs. The diagnostic yield of TBB was 76% on first rEBUS sessions. Forty-three (11%) out of 384 patients with initial non-diagnostic TBB procedures had a repeat procedure and were included for analysis (Fig. 1). The mean age of the study cohort was 66 ± 13 years and 58% were men. The average diameter of the PPLs was 36 ± 12 mm, and the localization of the lesions was the right upper lobe in 11 (26%) patients, right middle lobe in 10 (23%), right lower lobe in 7 (16%), left upper lobe in 8 (19%) and left lower lobe in 7 (16%). The majority of PPLs appeared solid (38/43, 88%) on CT images and the rEBUS probe was able to be positioned within in approximately three-fourths (32/43) of the lesions. The pathology of the first non-diagnostic rEBUS-guided TBB indicated atypical cells in 19 (44%) patients, normal lung tissue in 12 (28%), chronic inflammation in 9 (21%), and fibrosis, granulomatous inflammation and necrosis, each in 1 (2%). Repeat rEBUS-guided TBB was performed in an average of 12 days after the first procedure.

### Diagnostic yield of repeat TBB

A definitive diagnosis of PPLs was able to be achieved by repeat rEBUS-guided TBB in 23 (53%) patients. Table 1 shows the comparison between diagnostic and non-diagnostic PPLs on repeat rEBUS procedures. The diagnostic yield of repeat TBB was not affected by the size, location or CT appearance of the lesions, or position of the rEBUS probe. However, the pathology of the first rEBUS-guided TBB was significantly associated with the yield of repeat procedures (*P* = 0.011). Patients with normal lung tissue on initial pathology seldom (2/12, 17%) had a definite diagnosis on repeat rEBUS-guided TBB. Yet, patients with pathology showing atypical cells and other non-specific findings were more likely (21/31, 68%) to obtain a confirmed diagnosis by repeat TBB.

**Table 1:** Characteristics of the study population

	Repeat rEBUS-guided TBB		
Characteristic	Non-diagnostic	Diagnostic	*P* value
Patient No.	20	23	
Age, years	65 ± 15	66 ± 11	0.731
Male gender	13 (65)	12 (52)	0.395
Lesion size, mm	37 ± 10	35 ± 14	0.693
≤ 3 cm	4 (20)	7 (30)	0.434
˃3 cm	16 (80)	16 (70)	
CT appearance			
Solid	17 (85)	21 (93)	0.650
Non-solid	3 (15)	2 (8.7)	
Lesion location			
Upper lobes	9 (45)	10 (44)	0.920
Non-upper lobes	11 (55)	13 (57)	
Probe position			
Within	15 (75)	17 (74)	0.935
Adjacent to	5 (25)	6 (26)	
First pathology of TBB			
Normal lung tissue	10 (50)	2 (8.7)	0.011
Atypical cells	6 (30)	13 (57)	
Others	4 (20)	8 (35)	

*rEBUS* radial-probe endobronchial ultrasound, *TBB* transbronchial biopsy

The final diagnoses of PPLs are shown in Table 2. Malignancy, either primary or metastatic, was the diagnosis in the majority of study patients. Histopathologic characteristics of the lesions, benign (5/8, 63%) or malignant (17/35, 51%), did not affect the diagnostic yield of repeat rEBUS-guided TBB (*P* = 0.704).

**Table 2:** Final diagnosis of the peripheral pulmonary lesions

Category	Total *n* = 43
Malignancy	35 (81)
Adenocarcinoma (*n* = 22)	
Squamous cell carcinoma (*n* = 6)	
Metastasis (*n* = 3)	
Small cell carcinoma (*n* = 2)	
Non-small cell carcinoma (*n* = 1)	
Lymphoma (*n* = 1)	
Infection	4 (9.3)
Mycobacteriosis (*n* = 2)	
Cryptococcosis (*n* = 1)	
Pneumonia (*n* = 1)	
Other benign process	4 (9.3)

### Adverse events

Self-limited bleeding was observed in 3 patients on repeat TBB. One patient developed bleeding that required local hemostatic measures by using epinephrine spray. Another developed pneumothorax and was only treated with oxygen administration. No death occurred with the repeat rEBUS-guided procedures.

## Discussion

For the first time, this study shows the diagnostic yield of repeat rEBUS-guided TBB of PPLs after previous non-diagnostic procedures. A definitive diagnosis was able to be made in slightly over half (53%) of the repeat procedures. No clinical patient or lesion features were associated with the yield of repeat TBB. However, we found that the initial pathologic characteristics were predictive of the significance of repeat rEBUS-guided procedures. Patients with normal lung tissue on initial pathology were far less likely to have a diagnosis confirmed by repeat rEBUS-guided TBB than those with other pathologic findings. Regarding adverse events, bleeding and pneumothorax were seldom encountered. Taken together, the present study suggests that the overall diagnostic yield of repeat TBB was lower than the initial procedures; repeat procedures might not be a good choice for patients with initial pathology indicating normal lung tissue. Nonetheless, for other patients, repeat rEBUS-guided TBB may be a reasonable option for the diagnosis of PPLs after having previous non-diagnostic procedures (Table 3).

**Table 3:** Proposed indication for repeat rEBUS-guided TBB of PPLs

Proposed indication
Patients with PPLs and initial non-diagnostic rEBUS-guided TBB
Pathologic characteristics indicating findings other than normal lung tissue
Regardless of lesion size, CT appearance and location, and rEBUS probe position

*PPL* peripheral pulmonary lesion; *rEBUS* radial-probe endobronchial ultrasound; *TBB* transbronchial biopsy

Most of the time, patients with non-diagnostic rEBUS procedures for PPLs are scheduled for other invasive studies or follow-ups. Undoubtedly, repeat rEBUS-guided TBB is also an alternative to such studies or follow-ups, and is worth further investigation. We demonstrate herein that the diagnostic yield of repeat TBB (68%) may be comparable to that of first TBB (76%), if the target population for repeat procedures is confined to those who have had initial pathologic findings other than normal. On the other hand, patients with normal lung tissue on first pathology were not considered ideal candidates for repeat rEBUS-guided procedures, because a rather low diagnostic yield (17%) was observed. The possible explanation behind this intriguing finding could be intuitively simple. The presence of normal lung tissue on pathologic examination hypothetically indicates that there exists a barrier between the biopsy forceps and the target PPLs. Thus, it is expected that it remains difficult to obtain adequate tissue samples for diagnosis by using repeat rEBUS-guided TBB.

Lesion size, [9–11] probe position [8, 12, 13] and lesion histology [3] have all been shown to be associated with the diagnostic yield of rEBUS-guided TBB; however, the present study did not find such relationships. Our study population is composed of highly selected individuals, who have been chosen after initial non-diagnostic TBB, and based on the judgment of the physicians in charge. Needless to say, guidelines established from research regarding a more generalized population may not be applicable to our study cohort. Moreover, as the first study in this field, our results need to be validated in future work.

Safety is always an important issue in invasive diagnostic procedures. A barrage of evidence has proven that rEBUS-guided TBB is a secure modality for the diagnosis of PPLs [3, 4]. In line with prior observations, only 2 clinically significant adverse events, one each for bleeding and pneumothorax, occurred in this study, and they left no sequelae. Other diagnostic tools for PPLs are usually either more invasive (e.g. surgery) or more likely to incur complications (e.g. CT-guided biopsy), [14, 15] although they probably provide superior diagnostic yields [16]. In this regard, repeat rEBUS-guided TBB may be used as a second-line diagnostic procedure for PPLs after taking into account the advantages and disadvantages of choices on hand.

Our study carries a number of limitations. First, over a 5-year study period, we were only able to enroll a few patients; this may limit the statistical power of our study. However, we did bring out some significant findings and provide insight into this clinically important problem. Second, this was a retrospective study which did not include a control group to assess the efficacy of repeat rEBUS-guided TBB; without a doubt, the study design of our work did not allow us to comment on the best algorithm to confirm the diagnosis of PPLs following non-diagnostic rEBUS-guided TBB. Yet, to our knowledge, this is the first study to investigate the utility and safety of repeat TBB in this specific patient population. Hopefully, our results will encourage more large-scale and elaborate studies to further explore this issue. Third, this single-center experience may not be representative of broader practice patterns. Availability of a variety of diagnostic modalities, experience and preference of the practitioners, and disease characteristics and distribution may vary from institutions to institutions. Thus, in addition to pursuing multi-center collaboration and data generalizability, establishment of discrete, center-specific information is also of paramount importance. Finally, auxiliary bronchoscopic procedures, such as fluoroscopy or a guide sheath, were not conducted on our study subjects. Since adding these modalities to rEBUS-guided TBB may help achieve a rather favorable diagnostic yield in PPLs, [17, 18] it is uncertain whether our findings remain valid when ancillary tools are coupled to rEBUS-guided procedures.

## Conclusions

After initial non-diagnostic rEBUS-guided TBB, repeat procedures were capable of achieving a definitive diagnosis in approximately half of the PPLs. It is noteworthy that patients with normal lung tissue on first pathologic findings were unlikely to benefit from repeat TBB of the PPLs. On the other hand, the diagnostic yield of repeat rEBUS-guided procedures may be comparable to that of the first procedure in patients with initial pathology showing findings other than normal. The safety profile of repeat rEBUS-guided TBB in PPLs was equally favorable. Accordingly, the results of this study suggest that repeat TBB is a reasonable and viable option for the diagnosis of certain PPLs after previous non-diagnostic procedures.

## Abbreviations

PPL: Peripheral pulmonary lesion
rEBUS: Radial-probe endobronchial ultrasound
TBB: Transbronchial biopsy
